# Clinical significance of plasma PAF acetylhydrolase activity measurements as a biomarker of anaphylaxis: Cross-sectional study

**DOI:** 10.1371/journal.pone.0256168

**Published:** 2021-08-13

**Authors:** Krzysztof Łukasz Piwowarek, Agnieszka Rzeszotarska, Jolanta Łukasz Korsak, Aleksandra Juszkiewicz, Andrzej Chciałowski, Jerzy Kruszewski

**Affiliations:** 1 Department of Infectious Diseases and Allergology, Military Institute of Medicine, Warsaw, Poland; 2 Department of Transfusiology, Military Institute of Medicine, Warsaw, Poland; 3 Department of Internal Medicine and Rheumatology, Military Institute of Medicine, Warsaw, Poland; Mahidol Oxford Clinical Research Unitl (MORU), THAILAND

## Abstract

**Introduction:**

Platelet-activating factor (PAF) has a direct role as a mediator in the pathogenesis of various disorders with an inflammatory component, including those with allergic aetiology. The peripheral blood concentration of PAF is dynamically regulated by plasma PAF acetylhydrolase (PAF-AH). Previous research suggest that low activity of plasma PAF-AH could be a predictive marker for increased severity of some types of allergic hypersensitivity reactions–especially anaphylaxis. The purpose of the study was to evaluate the association between plasma PAF-AH activity and severity in patients with anaphylactic reactions following a wasp or bee sting.

**Materials and methods:**

The study group of 89 patients was divided into two subgroups depending on the increasing severity of the most severe anaphylactic reaction in the past, which was assessed according to the Müller’s scale. The first subgroup included participants with a history of hypersensitivity reactions up to grade II. The second subgroup consisted of patients who have experienced at least one grade III or IV reactions in the past. A control group of 20 people was established. Plasma PAF-AH activity was measured using a colorimetric method.

**Results:**

It has been observed that plasma activity of platelet-activating factor acetylhydrolase was significantly lower in patients with anaphylaxis history compared to the control group with negative atopic history (on average 21.38 nmol/min/ml for the control group, 9.47 nmol/min/ml for the first subgroup and 10.16 nmol/min/ml for the second subgroup, in both cases p < 0.0001).

**Conclusion:**

The plasma activity of PAF-AH is a promising parameter that can help to distinguish a group of patients not threatened with development of anaphylaxis and not requiring laborious or expensive prophylactic procedures.

## 1. Introduction

Platelet-activating factor (PAF), which was discovered in 1972 [1], is considered an important hypersensitivity mediator. Its influence includes anaphylaxis and its most severe form–anaphylactic shock. In the murine BALB/c model it was found, that PAF together with histamine may be responsible for the occurrence of anaphylaxis. The effect of PAF was more related to the reduction of cardiac output than to generalized vasodilation [2]. Moreover, some reports suggest that the importance of PAF as a mediator of anaphylaxis in humans may be superior to histamine and tryptase [3].

The concentration of PAF in the peripheral blood is regulated by plasma PAF acetylhydrolase (PAF-AH) [4, 5]. The physiological influence on this enzyme seems to be multidirectional [6, 7]. Previous studies indicate that the reduced activity of PAF-AH may be a potential risk factor not only for the occurrence but also for the severe course of food and *Hymenoptera* venom allergies (HVA) [8–10].

The aim of the study was to assess the relationship between the plasma PAF-AH activity and the severity of anaphylactic reaction after honeybee and wasp sting, towards a preliminary analysis of using this relationship for diagnostic purposes.

## 2. Materials and methods

### 2.1 Setting, study population, inclusion and exclusion criteria

The cross-sectional study was conducted in the Department of Infectious Diseases and Allergology and in the outpatient allergology clinic of the Military Institute of Medicine in Warsaw from December 2017 to April 2019.

All study participants had to be over 18 years old. The study group included patients who were consecutively admitted to the clinic due to an anaphylactic episode of varying severity after being stung by a honey bee or wasp. The episode should have occurred at least 21 days before enrolment in the study. The control group consisted of healthy people with negative atopic history. The exclusion criteria for both groups were identified as below:

1. The level of tryptase in the peripheral blood serum over 20 ng/ml.
2. History of congenital dyslipidemia.
3. History of mast cell dysfunction.
4. Acute disease.

### 2.2 Patients and controls

A group of 95 patients with HVA were qualified to participate in the study. The age range was from 22 to 76 years old. HVA was diagnosed in each case with standard intradermal tests or allergen-specific IgE blood test. 6 persons were disqualified due to the presence of exclusion criteria, resulting in a total of 89 patients included in the study.

The final study group of 45 women and 44 men, was then divided into two subgroups based on the Müller grading scale [11]: I-II, including participants with a history of hypersensitivity reactions up to grade II and III-IV, consisting of patients who have experienced at least one grade III or IV reaction in the past.

9 women and 11 men were enrolled in the control group. They were healthy and in a similar age range to the study group. They presented a negative history of allergic and non-allergic hypersensitivity reactions and did not meet the exclusion criteria.

### 2.3 Ethical issues

Military Institute of Medicine Bioethics Commission approval was obtained in accordance with the current revision of the Declaration of Helsinki [12]. Number of decision: 58/WIM/2016.

### 2.4 Data collection

After obtaining informed consent, the researcher interviewed the participants with a uniform questionnaire comprising of demographic and other data necessary for proper group qualification. The mentioned data include age, smoking status, concomitant diseases and history of anaphylactic reactions. Subsequently, basic anthropometric measurements were performed, such as weighting and height measurement. Serum tryptase levels were determined using the ImmunoCAP method.

Blood samples for the measurement of plasma PAF-AH activity were collected on citrate and transported on ice to the laboratory. Afterwards, the samples were centrifuged at 4°C for 10 minutes at an acceleration of 700 to 1000 g. The obtained plasma was stored frozen at -80°C. Finally, the authorized laboratory analyst defrosted samples and loaded into the PAF Acetylhydrolase Assay Kit item No. 760901 produced by Cayman Chemical Company (USA). The test, based on the colorimetric method, was performed according to the manufacturer’s instructions. We used radiation with a wavelength of 412 nm. Measurements of the absorbance were made six times in a row, at 1-minute intervals in order to plot the appropriate linear function. The plasma PAF-AH activity was calculated in units of nmol/min/ml using a properly modified formula provided by the manufacturer of the kit.

### 2.5 Statistical analysis

All statistical tests were performed using the STATISTICA 13.0 software provided by StatSoft Inc. (Poland). The Kolmogorov-Smirnow test was used to determine the significance of differences in the distribution of the studied variables in relation to the normal distribution. Student’s t-test was used for the comparison of means from the groups with a normal distribution, while for others the Mann-Whitney U test was used. For comparisons of multiple groups, the Bonferroni correction for the t-test and Kruskal-Wallis ANOVA rank test were used, respectively.

Categorical variables were presented as n (%) and compared with each other using the χ^2^ test. The control group and research subgroups were paired to test the null hypothesis that plasma PAF-AH activity is not related to the severity of the post-stinging reaction. The established level of statistical significance was α = 0.05.

The correlations between the variables were investigated by calculating the Spearman’s rank coefficient. It was considered significant when: r > 0.3 or r < -0.3.

A regression analysis was performed and the receiver operating characteristic (ROC) curve was laid out. Typical test parameters were calculated according to well-known definitions, such as sensitivity, specificity, accuracy, positive predictive value and negative predictive value [13].

## 3. Results

### 3.1 Population characteristics

As shown in Tables 1 and 2, subgroups I-II included 8 patients with a history of the first-grade reaction and 31 patients after second-grade reactions. Subgroups III-IV included 33 patients after third-grade reactions and 17 patients after fourth-grade reactions, respectively.

**Table 1:** Characteristics of the study subgroups—continuous variables.

Variable	Subgroup I-II			Subgroup III-IV			p	Control group n = 20			p	
	n = 39			n = 50								
	Mean	SD	Median	Mean	SD	Median		Mean	SD	Median	vs I-II	vs III-IV
**Age [years]**	**48,26**	12,20	46,00	**45,36**	13,88	43,50	0,2301	**44,15**	13,33	45,00	0,2810	0,9950
**Body mass [kg]**	**81,95**	17,99	85,00	**76,56**	15,16	75,00	0,1160	**79,50**	12,74	80,00	0,6637	0,4181
**Height [cm]**	**171,86**	9,86	171,00	**171,02**	9,09	170,00	0,5700	**175,44**	11,49	174,50	0,3528	0,1907
**BMI [kg/m** ^**2**^ **]**	**27,59**	5,03	27,14	**26,05**	4,01	25,46	0,1002	**25,84**	3,29	26,44	0,3023	0,7725
**Tryptase [ng/ml]**	**5.59**	4.13	3.94	**4.77**	2.69	4.04	0.8167	**5.46**	2.53	4.89	0.2878	0.1465
	[1.67; 17.6]			[1; 13.1]				[2.15; 11.3]				

**Table 2:** Comparison of subgroups within the study population regarding plasma PAF acetylhydrolase activity.

Comparised series	Mean [nmol/min/ml]	SD	Median	Test	p
**Control group**	21,38	9,50	25,59	U Manna-Whitney	0,000001
**Subgroup I-II**	9,47	1,87	9,15		
**Control group**	21,38	9,50	25,59	U Manna-Whitney	0,000014
**Subgroup III-IV**	10,16	2,50	9,85		
**Subgroup I-II**	9,47	1,87	9,15	t Student	0,060902
**Subgroup III-IV**	10,16	2,50	9,85		
**Control group**	21,38	9,50	25,59	Kruskal-Wallis test	0,0001
**1st grade of severity**	8,93	2,13	8,97		
**2nd grade of severity**	9,61	1,80	9,23		
**3rd grade of severity**	10,24	2,64	9,67		
**4th grade of severity**	10,01	2,26	9,94		

No statistically significant differences were found between subgroups I-II, III-IV and the control group in terms of basic demographic, anthropometric and biochemical data. In the both studied subgroups, IgE-dependent reaction to wasp venom was a more frequent cause of post-stinging reactions than allergy to honey bee venom. Therefore, differences were statistically insignificant.

### 3.2 Plasma PAF-AH activity

**Fig 1:**
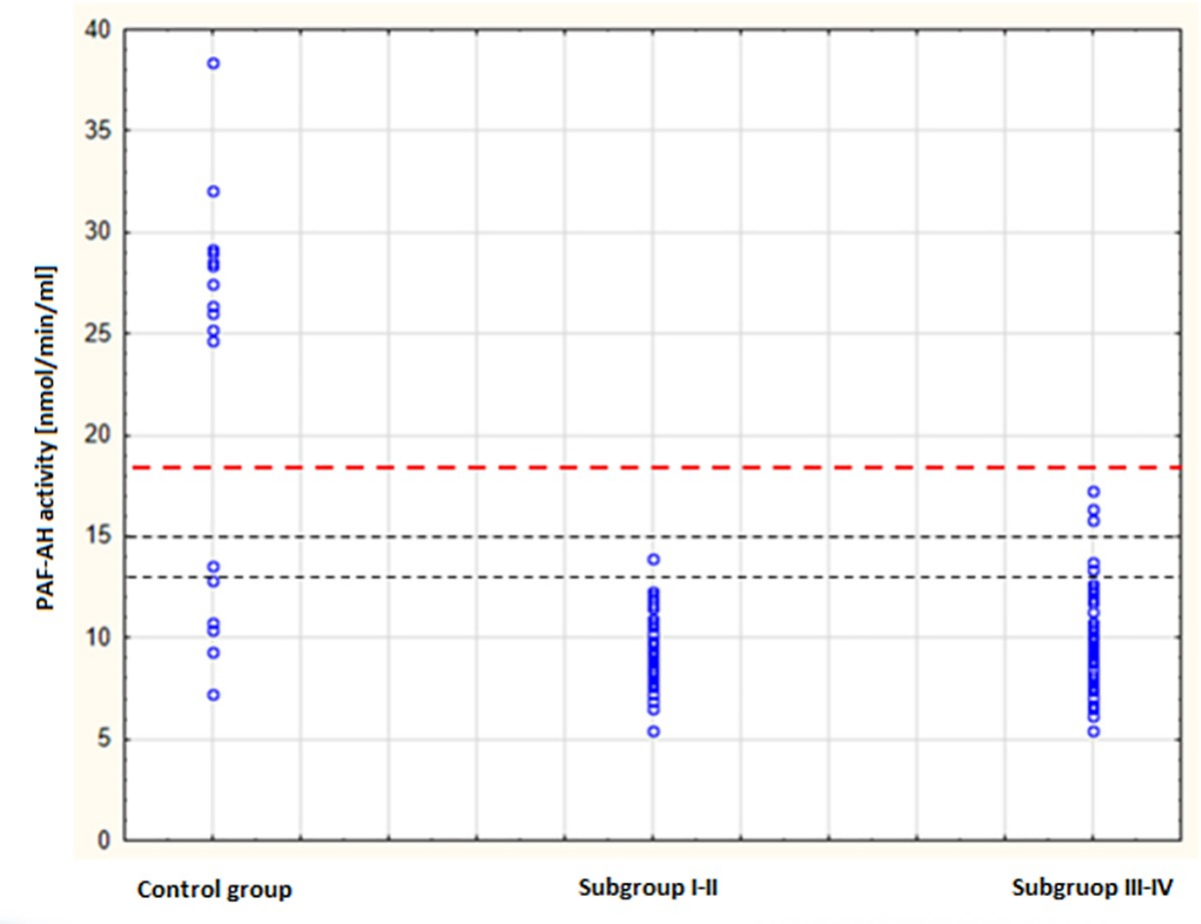
Scatter plot of PAF-AH plasma activity by severity of anaphylaxis.

The distributions of PAF-AH activity in the study group and the control group are presented in Fig 1. They differed substantially, which was reflected in the highly statistically significant difference in the arithmetic means in both groups.

**Fig 2:**
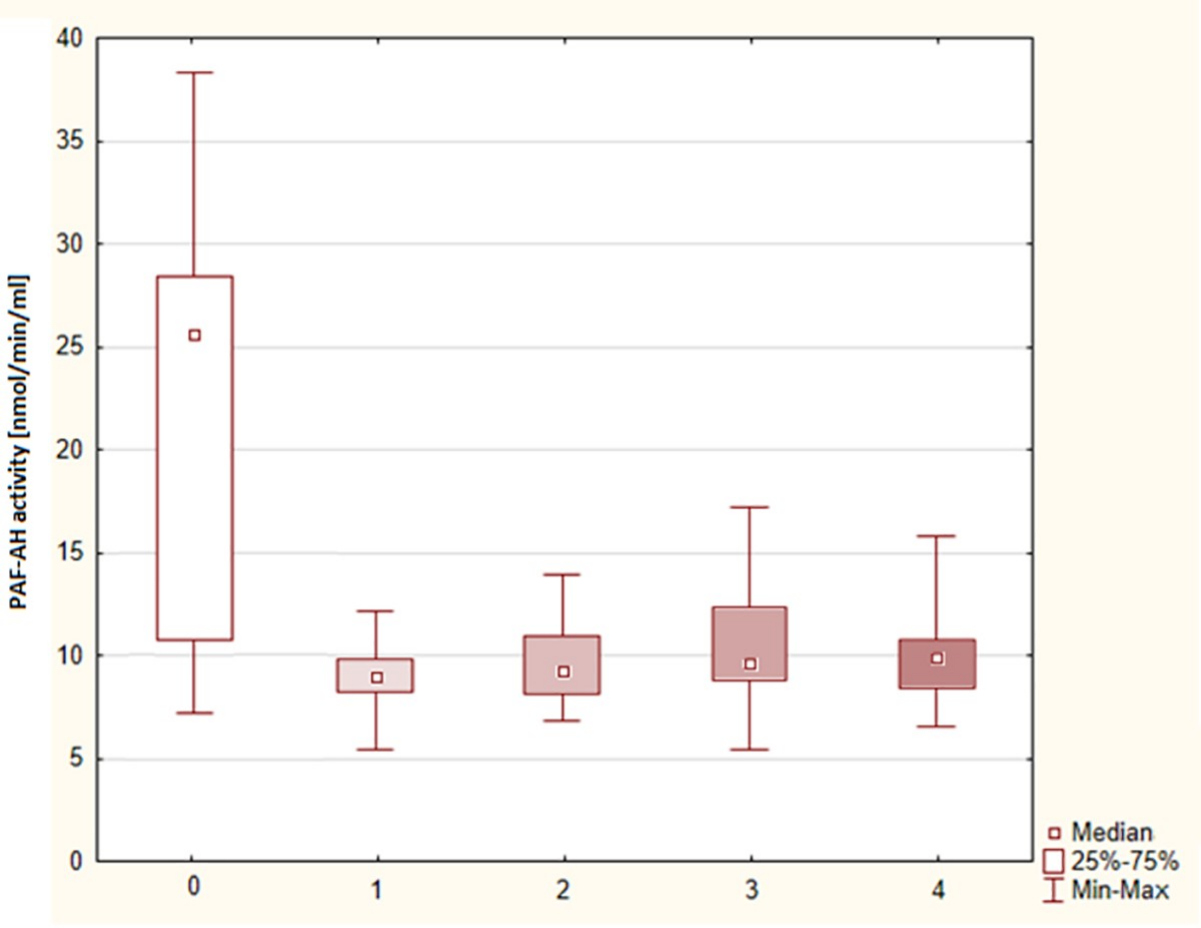
Distribution of PAF-AH plasma activity by severity of anaphylaxis–box plot. 0 –control group, 1, 2, 3, 4 –grade of severity according to Müller’s classification.

As shown in Fig 2, no significant differences were found in the arithmetic mean values of plasma PAF-AH activity in the individual grades of severity of anaphylaxis (p > 0.05). Although, these values were considerably lower than the value in the control group, which was 21.38 ± 9.50 nmol/min/ml (p < 0.0001).

According to Table 2, the arithmetic mean values of plasma PAF-AH activity in the subgroup I-II (9.47 ± 1.87 nmol / min / ml) and in the subgroup III-IV (10.16 ± 2.50 nmol/min/ml) also did not differ significantly (p > 0.05) but were substantially lower as compared to the value in the control group (p < 0.0001).

The arithmetic mean values of plasma PAF-AH activity in the smokers and non-smokers did not differ significantly (9.46 ± 1.95 nmol/min/ml vs 10.13 ± 1.61 nmol/min/ml, p = 0.201).

There was no statistically significant correlation between the plasma PAF-AH activity and most of the parameters, including the age of the patients (r = -0.035). There was only a statistically significant, but clinically irrelevant, correlation with tryptase concentration (r = 0.21).

### 3.3 The performance of PAF-AH activity measurements in recognizing anaphylaxis

**Fig 3:**
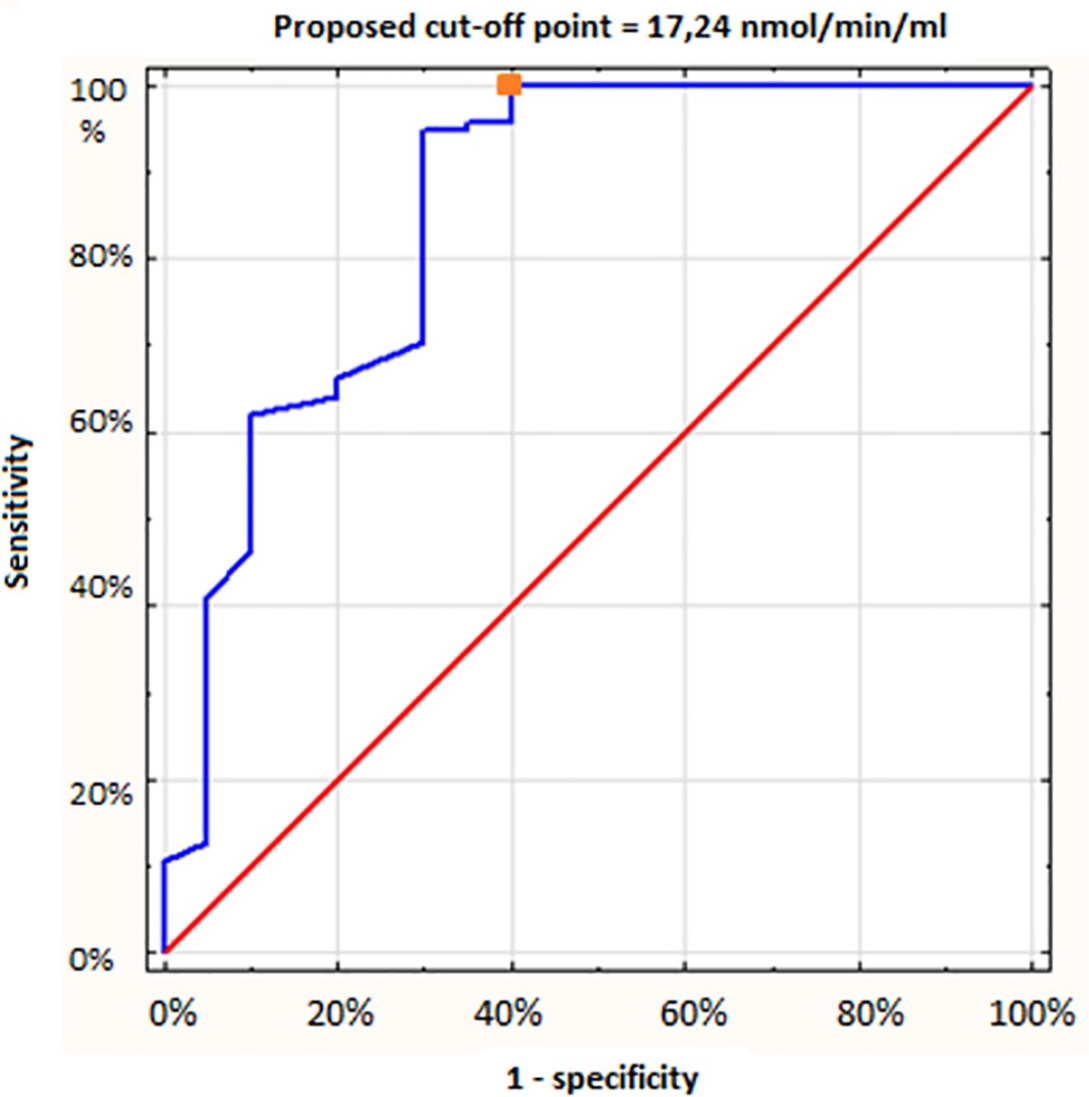
ROC for PAF-AH plasma activity test.

The result of the regression analysis is shown in Fig 3. The optimal cut-off point for PAF-AH activity to discriminate anaphylactic and non-anaphylactic patients was 17.24 nmol/min/ml at the following parameters:

- Sensitivity: 100%
- Specificity: 60%
- Accuracy: 93%
- Positive predictive value (PPV): 92.2%
- Negative predictive value (NPV): 100%
- Misclassification rate: 7%
- Area under the curve (AUC): 0.854
- Slope factor: 0.21

## 4. Discussion

In the presented study it was confirmed that the mean activity of plasma PAF-AH activity is significantly lower in patients with a history of anaphylaxis in the course of HVA as compared to healthy people. The individual’s specific predisposition resulting from the enzyme activity may also play an important role in the pathogenesis of disorder.

Contrary to other authors, our research did not show an obvious correlation between PAF-AH activity and the severity of the hypersensitivity reaction to the venom [10]. This problem has been studied and discussed since the early 2000s. Vadas et al. analyzed the relationship between plasma PAF-AH activity and the severity of anaphylaxis in children and adult patients [8, 9, 14]. Reactions were triggered by foods, medications or insect stings. In the prospective part of the mentioned authors study, three groups with increasing severity of anaphylaxis were recruited. The control was a group of non-allergic children. In contrast to our study, it showed a trend suggesting that decreased PAF-AH activity is associated with a more severe course of an anaphylactic reaction. However, statistical significance was not obtained.

A statistically significant association was found in the retrospective section of the study. It compared 9 cases of fatal peanut anaphylactic reactions to several other groups, including children who died from non-anaphylactic causes, non-allergic children, and children with urticaria or peanut-induced angioedema. In all these comparisons, the plasma PAF-AH activity was significantly lower in children who died from anaphylaxis. [8, 9].

In subsequent years, the same researchers found that patients with the lowest levels of plasma PAF-AH activity had a 27-fold higher risk of severe or fatal anaphylaxis. Finally, Vadas et al. found a correlation between PAF-AH activity and the age of the participants, but in our study, it was lacking.

Research on the plasma PAF-AH and food anaphylaxis was also conducted in the Moscow study groups of children with a history of food anaphylaxis, atopic dermatitis and a negative history of allergic diseases. It was found that children with a history of food anaphylaxis had statistically lowered level of PAF-AH mRNA in peripheral blood compared to both other groups of children. In the group of food anaphylaxis, lower expression of the PAF-AH gene was associated with a more severe course of anaphylaxis and cardiovascular symptoms [15].

The relationship between plasma PAF-AH activity and the severity of anaphylactic reactions to Hymenoptera venom has been investigated and discussed previously and was no statistically significant difference in PAF-AH activity between healthy controls and patients after the mild anaphylactic reactions of the 1^st^ grade of severity. The subgroups with higher severity grades had significantly and stepwise lower mean plasma PAF-AH activity compared to the control group and to each other. The exception has occurred for the 3rd and 4th severity subgroups, between which the difference was statistically irrelevant. Logistic regression showed that only PAF-AH activity was significantly associated with the severity of anaphylaxis. The best cut-off point for grades II-IV was 25 nmol/ml/min [10].

The outcomes of our trial differ from the results of the presented studies. The difference in the mean activity of the enzyme between the control group and the study subgroups was more than twofold. The highest PAF-AH activity in a patient with a positive history of anaphylaxis was only 17.24 nmol/min/ml. The cut-off point of 20 nmol/min/mL proposed by Pravettoni et al. discriminates patients who are not at risk of developing severe anaphylaxis very well, but our study suggests the possibility of further lowering this threshold. Notwithstanding, the most intriguing is the combination of two study subgroups—grade I-II and III-IV. They did not differ in terms of enzyme activity (9.47 vs 10.16 nmol/min/ml, p = 0.0609 in Student’s t-test). Therefore, we did not find a relationship between the severity of anaphylactic reaction in the past and the plasma PAF-AH activity. This is interesting in the context of the studies by Pravettoni et al., which show this kind of association.

There are several potential reasons for the above-mentioned divergence. Apart from the various size of the samples, age of the participants and distinct local practice in qualifying patients for the different severity grades of anaphylaxis, the discussed studies differed in design. Unlike the first Vadas studies, it seems that our study group comprised patients with less severe reactions, e.g. we did not investigate cases of fatal anaphylaxis. The study performed by Pravettoni et al. concerned only newly diagnosed patients, while our study did not exclude patients undergoing immunotherapy. Technical aspects affecting the results may also be important. In the Italian study, 405 nm light was used to measure absorbance, while in our trial wavelength was 412 nm.

Irrespective of the presented explanations, the results of our study suggesting the participation of PAF-AH in the pathogenesis of anaphylaxis lead to the consideration of the practical application of the measurements of plasma PAF-AH. In our population, all patients with a history of anaphylaxis had decreased PAF-AH activity. This confirms that the determination of PAF-AH activity can be a particularly useful tool in the stratification of the individual risk of anaphylaxis to *Hymenoptera* venoms, especially in beekeepers. Assuming that the clinical effect of stinging, on the one hand, depends on the strength of the anaphylactic effect of the venom, and on the other hand on the individual properties of the sensitized person, the measurement of PAF-AH plasma activity allows us to assess a significant internal factor influencing the occurrence of symptoms. Perhaps the test could also help in the diagnosis of non-allergic and idiopathic anaphylaxis—distinguishing the true anaphylaxis from numerous conditions with similar symptoms, such as somatoform disorders. Presently, the only laboratory test widely used in such cases is the measurement of tryptase concentration during an acute episode, which is often impossible to perform for many reasons. The measurement of plasma PAF-AH activity could provide a lot of diagnostic information, even months or years after an anaphylaxis episode.

Unfortunately, our study plan did not take into account the group of patients with other severe forms of hypersensitivity without a history of an anaphylactic reaction. This would allow us to answer whether the decreased PAF-AH activity occurs only in patients with anaphylaxis, or in general in patients with severe hypersensitivity to one or more factors. Also, we did not consider the group of patients suspected of hereditary alpha tryptasaemia, which can affect the severity of anaphylaxis.

A relatively wide dispersion of the results of plasma PAF-AH activity was found in the control group. Noteworthy, this group was selected primarily on the basis of negative atopic history. It is impossible to predict a priori whether some of these people will be at risk of developing anaphylaxis in the future. Perhaps currently healthy individuals with low PAF-AH activity could be considered capable of developing an anaphylactic reaction in the future. The distribution of results in the control group may also suggest the presence of PAF-AH gene polymorphisms in the Polish population, which strongly affect the enzyme activity. These problems will be the subject of further research.

## 5. Conclusions

- The plasma activity of PAF-AH is a promising parameter that can help to distinguish a group of patients not threatened with development of anaphylaxis and not requiring laborious or expensive prophylactic procedures.
- Reduced plasma PAF-AH activity below 17 nmol/min/ml is characteristic of patients who develop anaphylactic reactions after an insect sting.
- Contrary to expectations, no significant correlation was found between the degree of impairment of plasma PAF-AH activity and the grade of severity of anaphylaxis following an insect sting.

## Supporting information

S1 File Survey–participant information. (DOCX)
