# Supplementary material for: Clinical significance of plasma PAF acetylhydrolase activity measurements as a biomarker of anaphylaxis: Cross-sectional study
Source: PLoS One. 2021 Aug 13;16(8):e0256168. doi: 10.1371/journal.pone.0256168 (PMC8362976; doi:10.1371/journal.pone.0256168)
Supplement: S1 File — (DOCX) [file pone.0256168.s001.docx]

| **Participant Information** | |
| --- | --- |
| **Participant number** |  |
| **Name and surname** |  |
| **Date of birth** |  |
| **Age** |  |
| **ID number** |  |
| **Gender** | Male / Female |
| **Pregnant** | Yes / No |
| **Date of enrollment** |  |
| **Date of informed consent** |  |
| **History of congenital dyslipidemia** | Yes / No |
| **History of mast cell dysfunction** | Yes / No |
| **Acute disease** | Yes / No |
| **Had anaphylaxis in the last 21 days** | Yes / No |
| **Smoking** | Yes / No |
| **Allergic rhinitis** | Perennial / Seasonal / No |
| **Asthma** | Atopic / Non-atopic / No |
| **Urticaria** | Yes / No |
| **Bee sting anaphylaxis** | Yes / No |
| **Wasp sting anaphylaxis** | Yes / No |
| **Drug anaphylaxis** | Yes / No |
| **Food anaphylaxis** | Yes / No |
| **Grade of the most severe anaphylaxis** | I / II / III / IV |
| **Arterial hypertension** | Yes / No |
| **Coronary artery disease** | Yes / No |
| **Diabetes** | Yes / No |
| **COPD** | Yes / No |
| **Statin user** | Yes / No |
| **Body mass** |  |
| **Height** |  |
| **Systolic blood pressure** |  |
| **Diastolic blood pressure** |  |

| **Kwestionariusz uczestnika** | |
| --- | --- |
| **Numer uczestnika** |  |
| **Imię i nazwisko** |  |
| **Data urodzenia** |  |
| **Wiek** |  |
| **PESEL** |  |
| **Płeć** | Mężczyzna / Kobieta |
| **Ciąża** | Tak / Nie |
| **Data włączenia** |  |
| **Data podpisania świadomej zgody** |  |
| **Wrodzona dyslipidemia** | Tak / Nie |
| **Dysfunkcja komórek tucznych** | Tak / Nie |
| **Ostra choroba** | Tak / Nie |
| **Anafilaksja w ciągu ostatnich 21 dni** | Tak / Nie |
| **Palenie** | Tak / Nie |
| **Alergiczny nieżyt nosa** | Całoroczny / Sezonowy / Nie |
| **Astma** | Atopowa / Nieatopowa / Nie |
| **Pokrzywka** | Tak / Nie |
| **Anafilaksja na jad pszczoły** | Tak / Nie |
| **Anafilaksja na jad osy** | Tak / Nie |
| **Anafilaksja polekowa** | Tak / Nie |
| **Anafilaksja pokarmowa** | Tak / Nie |
| **Stopień ciężkości anafilaksji** | I / II / III / IV |
| **Nadciśnienie tętnicze** | Tak / Nie |
| **Choroba wieńcowa** | Tak / Nie |
| **Cukrzyca** | Tak / Nie |
| **POChP** | Tak / Nie |
| **Stosowanie statyn** | Tak / Nie |
| **Masa ciała** |  |
| **Wzrost** |  |
| **Ciśnienie tętnicze skurczowe** |  |
| **Ciśnienie tętnicze rozkurczowe** |  |
